# Molecular basis of autotrophic vs mixotrophic growth in *Chlorella sorokiniana*

**DOI:** 10.1038/s41598-018-24979-8

**Published:** 2018-04-24

**Authors:** M. Cecchin, S. Benfatto, F. Griggio, A. Mori, S. Cazzaniga, N. Vitulo, M. Delledonne, M. Ballottari

**Affiliations:** Dipartimento di Biotecnologie, Università di Verona, Strada Le Grazie 15, 37134 Verona, Italy

## Abstract

In this work, we investigated the molecular basis of autotrophic vs. mixotrophic growth of *Chlorella sorokiniana*, one of the most productive microalgae species with high potential to produce biofuels, food and high value compounds. To increase biomass accumulation, photosynthetic microalgae are commonly cultivated in mixotrophic conditions, adding reduced carbon sources to the growth media. In the case of *C*. *sorokiniana*, the presence of acetate enhanced biomass, proteins, lipids and starch productivity when compared to autotrophic conditions. Despite decreased chlorophyll content, photosynthetic properties were essentially unaffected while differential gene expression profile revealed transcriptional regulation of several genes mainly involved in control of carbon flux. Interestingly, acetate assimilation caused upregulation of phosphoenolpyruvate carboxylase enzyme, enabling potential recovery of carbon atoms lost by acetate oxidation. The obtained results allowed to associate the increased productivity observed in mixotrophy in *C*. *sorokiniana* with a different gene regulation leading to a fine regulation of cell metabolism.

## Introduction

Photosynthetic conversion of light provides the energy necessary for biomass formation in living organisms. Among photosynthetic species, unicellular microalgae are of great interest due to their high potential for industrial cultivation as light energy converting systems for the production of biomass, food and biofuels, without being in competition with traditional agriculture^1,2^. Photo-autotrophic growth of microalgae indeed requires light, CO_2_, water and nutrients yielding lipids, proteins and sugars rich biomass. However, some microalgae species have also the peculiar capability to grow in mixotrophic mode, where the autotrophic metabolism is integrated with a heterotrophic metabolism, that oxidizes the reduced carbon source available in the medium. Mixotrophic cultivation of microalgae holds the potential to significantly improve biomass production, thus fostering the revenues of industrial cultivation: this is particularly important for biofuels production, where productivity and cultivation costs must be respectively maximized and minimized to be sustainable^1,3^. The main substrates used for mixotrophic growth of microalgae are glucose, ethanol or cheaper waste products of several industrial processes as acetate or glycerol. Extensive work on *Chlamydomonas reinhardtii*, the model organism for green algae, demonstrated increased biomass and lipid productivity in mixotrophy compared to autotrophy^4,5^. Even in the case of non-model species as *Chlorella spp*. or *Scenedesmus*, mixotrophic growth is effective to increase the biomass and lipid productivity^3,6,7^. However, this is not a general feature of microalgae, since some species, as the marine algae *Nannochloropsis gaditana*, exhibited similar growth in autotrophy and in presence of different reduced carbon source, due to a reduced photosynthetic efficiency in mixotrophy^8^. In this work autotrophic growth of the thermotolerant high productive strain *Chlorella sorokiniana* was compared to its mixotrophic growth in the presence of acetate as reduced carbon source in the medium. Acetate was reported to be assimilated in *C*. *reinhardtii* as acetyl-CoA which enters the Krebs cycle upon condensation with oxaloacetate to produce citrate. Acetate assimilation in several algae species is strictly linked to the activity of isocitrate lyase enzyme which redirects isocitrate toward the glyoxylate cycle, thus preventing carbon loss as CO_2_ upon completion of the Krebs cycle^6,9–11^. Acetyl-CoA is mainly produced in photosynthetic organisms by oxidation of pyruvate mediated by the mitochondrial pyruvate dehydrogenase enzyme and fueled by photosynthetic produced sugars. An alternative pathway for acetate assimilation is present in the chloroplast, where acetyl-CoA is used for *de novo* production of fatty acids^12^. The interaction between photosynthesis and acetate metabolism is further complicated by the reciprocal influence of mitochondria and chloroplasts redox state: for instance, in *C*. *reinhardtii* alteration of mitochondrial respiration and different NADH availability changed the NADPH content in the chloroplast, inducing different reduction state of plastoquinones^4,13^. To investigate the influence of mixotrophic growth in presence of acetate in *C*. *sorokiniana*, photosynthetic properties and differential gene expression of autotrophic *vs*. mixotrophic cultures were thus analyzed in order to identify strategy to further foster the metabolism and improve biomass production for industrial applications.

## Results

### Cultivation of C. sorokiniana in autotrophy vs. mixotrophy

**Figure 1:**
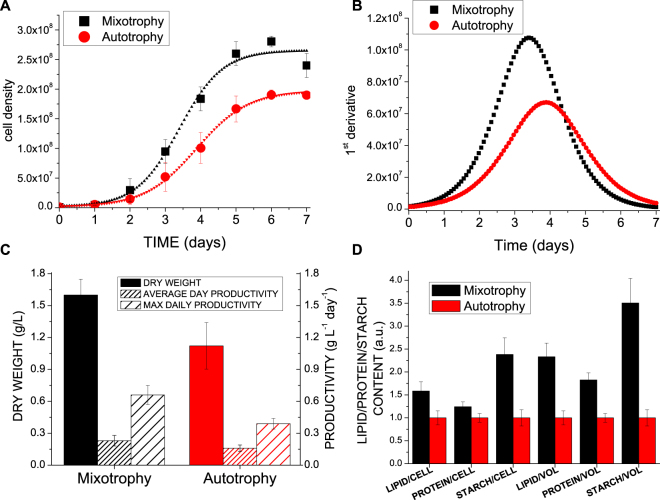
Growth curves, biomass productivity and accumulation of macromolecules in mixotrophy *vs*. autotrophy. Panel A: growth curves of *C*. *sorokiniana* growth in autotrophy *vs*. mixotrophy fitted with sigmoidal curves; Panel B: first derivate of growth curves reported in Panel A; Panel C: dry weight and average daily productivity; Panel D: relative protein, lipid and starch content per cell or volume of culture.

*C*. *sorokiniana* cells were grown in airlift photobioreactors in presence or absence of acetate, inducing respectively mixotrophic or autotrophic metabolism. Cells grown in mixotrophy reached higher cell density compared to cells grown in autotrophy, with a 61% increase in the presence of acetate (Fig. 1A). Growth curves were fitted with sigmoidal functions with lower dx values in mixotrophy (0.61) compared to autotrophy (0.73) indicating faster growth triggered by acetate. Moreover, mixotrophic growth was characterized by the highest daily maximum productivity estimated from the first derivate of the sigmodial fitting functions (Fig. 1B). Mixotrophy determined 42% increase of total biomass production as compared to autotrophy, with an average and maximum daily productivity of 0.23 and 0.66 gr L^−1^day^−1^, respectively, in mixotrophy and 0.16 and 0.39 gr L^−1^day^−1^ in autotrophy (Fig. 1C). The productivity obtained in autotrophic conditions allowed to estimate a photosynthetic efficiency of ~2.36% on the basis of average daily productivity and ~5.86% considering the maximum daily productivity. In mixotrophy, 2 Kcal/L increase of energy stored as biomass was obtained as a consequence of the 3.48 Kcal/L added to the growth medium as acetate, indicating a metabolic energetic efficiency of acetate utilization of at least ~57%, close to the energetic yield of acetyl-CoA oxidized by the Krebs cycle previously reported in the case of *C*. *reinhardtii*^5^. As expected lipids, proteins and starch were all consistently increased in mixotrophy-grown cells when compared to autotrophic conditions, of 58%, 24% and 138% respectively on a cell basis. Considering the higher cell density reached in mixotrophy compared to autotrophy, the lipid, protein and starch productivity per volume of culture were increased in mixotrophy by ~133%, ~82% and ~250% respectively (Fig. 1D).

### Photosynthetic properties of C. sorokiniana in autotrophy vs. mixotrophy

**Figure 2:**
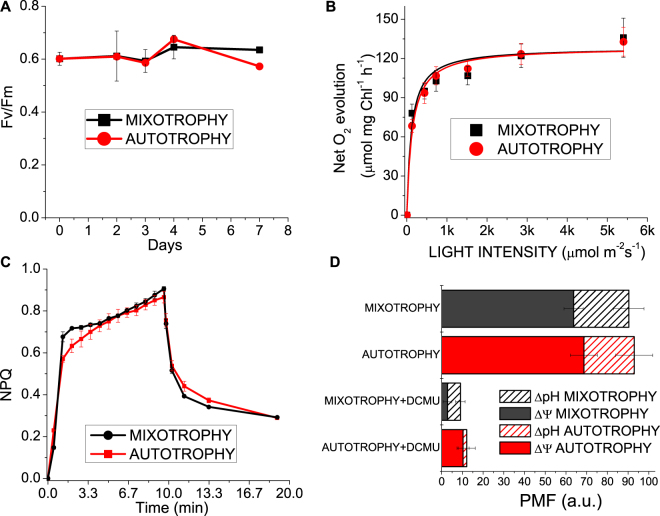
Photosynthetic properties of *C*. *sorokiniana* in autotrophy vs. mixotrophy. Panel A: PSII efficiency (Fv/Fm) variation during cultivation; Panel B: net oxygen evolution curves at different light intensities fitted with hyperbolic functions; Panel C: NPQ induction curves measured at 1500 µmol m^−2^s^−1^. Panel D: proton motive force (PMF) induced by 940 µmol m^−2^s^−1^ in autotrophic and mixotrophic cells. Chemical (ΔpH) and electric (ΔΨ) components of PMF are indicated.

Photosynthetic properties of *C*. *sorokiniana* in autotrophy *vs*. mixotrophy were investigated to determine the influence of acetate assimilation on the autotrophic metabolism. *C*. *sorokiniana* cells was characterized by a ~50% reduction of chlorophyll content per cell in mixotrophy compared to autotrophy (Supplementary Table S1). Chl a/b and Chl/Car ratios however were similar in both conditions and the carotenoid composition was not significantly different when normalized to chlorophyll content. The fluorescence parameter Fv/Fm is generally used as an indicator of the wellness of the photosynthetic apparatus, being related to the photochemical efficiency of the PSII. As reported in Fig. 2A, cells grown either in autotrophic or mixotrophic conditions showed similar Fv/Fm values of ~0.6 through the entire cultivation period. Net oxygen evolution curves measured at different light intensities were similar on a chlorophyll basis for cells grown in autotrophy or mixotrophy (Fig. 2B) and the parameters Pmax and half saturation light intensities, calculated by hyperbolic fitting of the oxygen evolution curves, were not significantly different (Supplementary Table S2). However, dark respiration measured on mixotrophic cells was increased compared to autotrophic cells, either when normalized per chlorophyll content, or per cell concentration (Supplementary Table S2) indicating increase mitochondrial electron transport to oxygen as a consequence of increased NADH production in the presence of acetate. The photoprotective properties of *C*. *sorokiniana* cells were then investigated measuring the Non-photochemical quenching (NPQ) induction curves: this process consists into thermal dissipation of a variable portion of the light absorbed by the photosynthetic apparatus and it is induced upon lumen acidification when light is absorbed in excess. As reported in Fig. 2C, NPQ curves were almost identical in autotrophic or mixotrophic cells, demonstrating that *C*. *sorokiniana* has similar photoprotective properties in the two conditions. NPQ induction is triggered by lumen acidification: total proton motive force (PMF) and its composition in chemical (ΔpH) and electric (ΔΨ) components were thus analyzed by pump-probe transient absorption at 520 nm, since carotenoids shift their absorption when electrochemical proton gradient across thylakoid membranes is established (Electro-Chromic Shift ECS)^14^. PMF ΔpH and ΔΨ were not significantly different in mixotrophy compared to autotrophy (Fig. 2D). When linear photosynthetic electron transport was blocked adding the PSII inhibitor DCMU, a strong reduction in PMF was observed in both conditions, with a higher ΔpH formation in mixotrophy compared to autotrophy. This result indicates an increased PSII-independent reduction of plastoquinones in the presence of acetate which oxidation causes release of protons, as reported in the case of *C*. *reinhardtii*^10,13^.

### De novo transcriptome assembly and gene expression analysis of C. sorokiniana in autotrophy vs. mixotrophy

**Figure 3:**
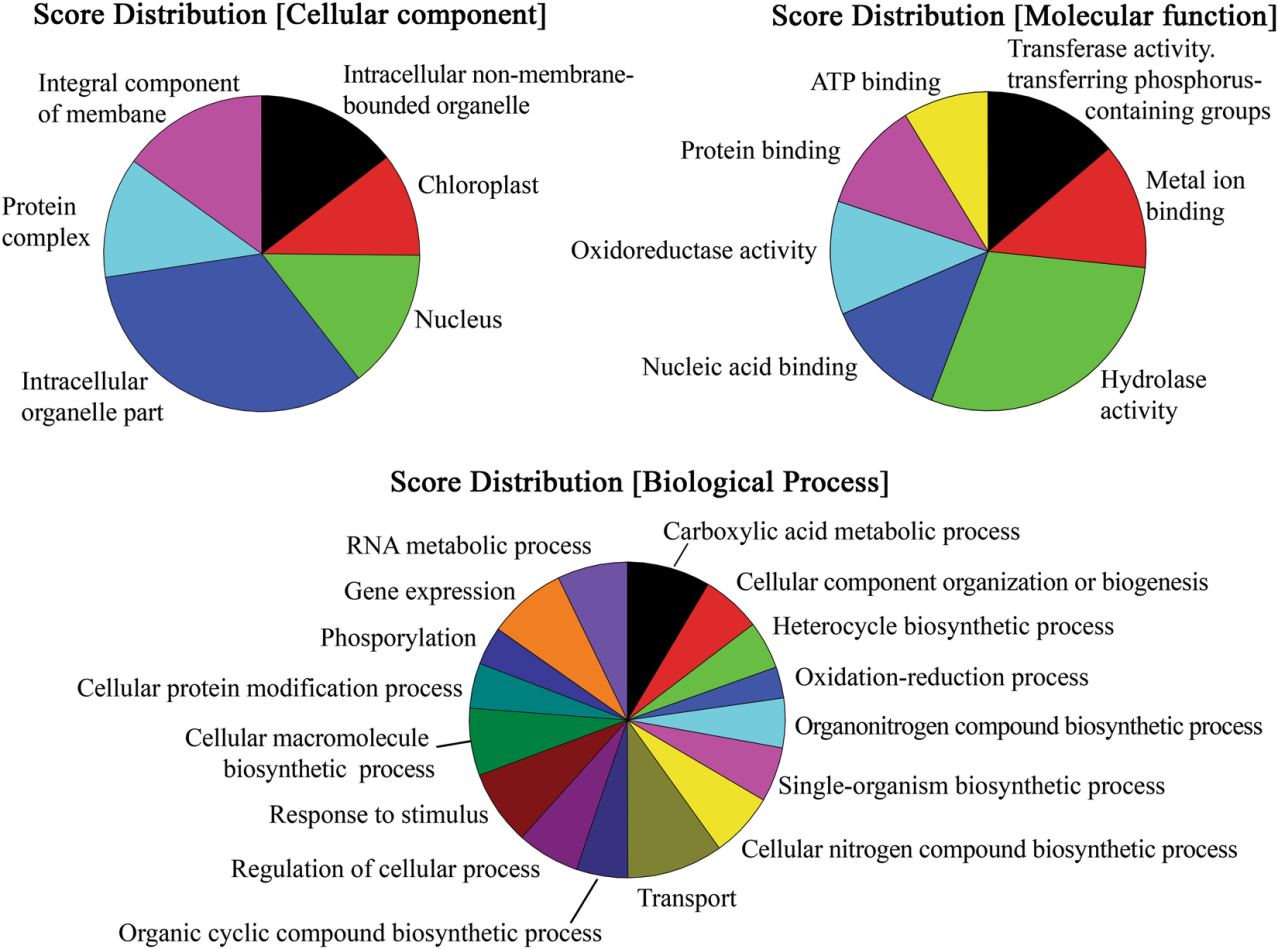
Annotation of *C*. *sorokoniana* transcriptome. *C*. *sorokiniana* transcripts annotated by blast2Go were functionally grouped on the basis of Gene Onthology (GO) terms “cellular component”, “molecular function” and “biological processes”. The distribution of the different groups is reported on the basis of the node score associated to each group considering GO term with node score higher than 1%.

**Figure 4:**
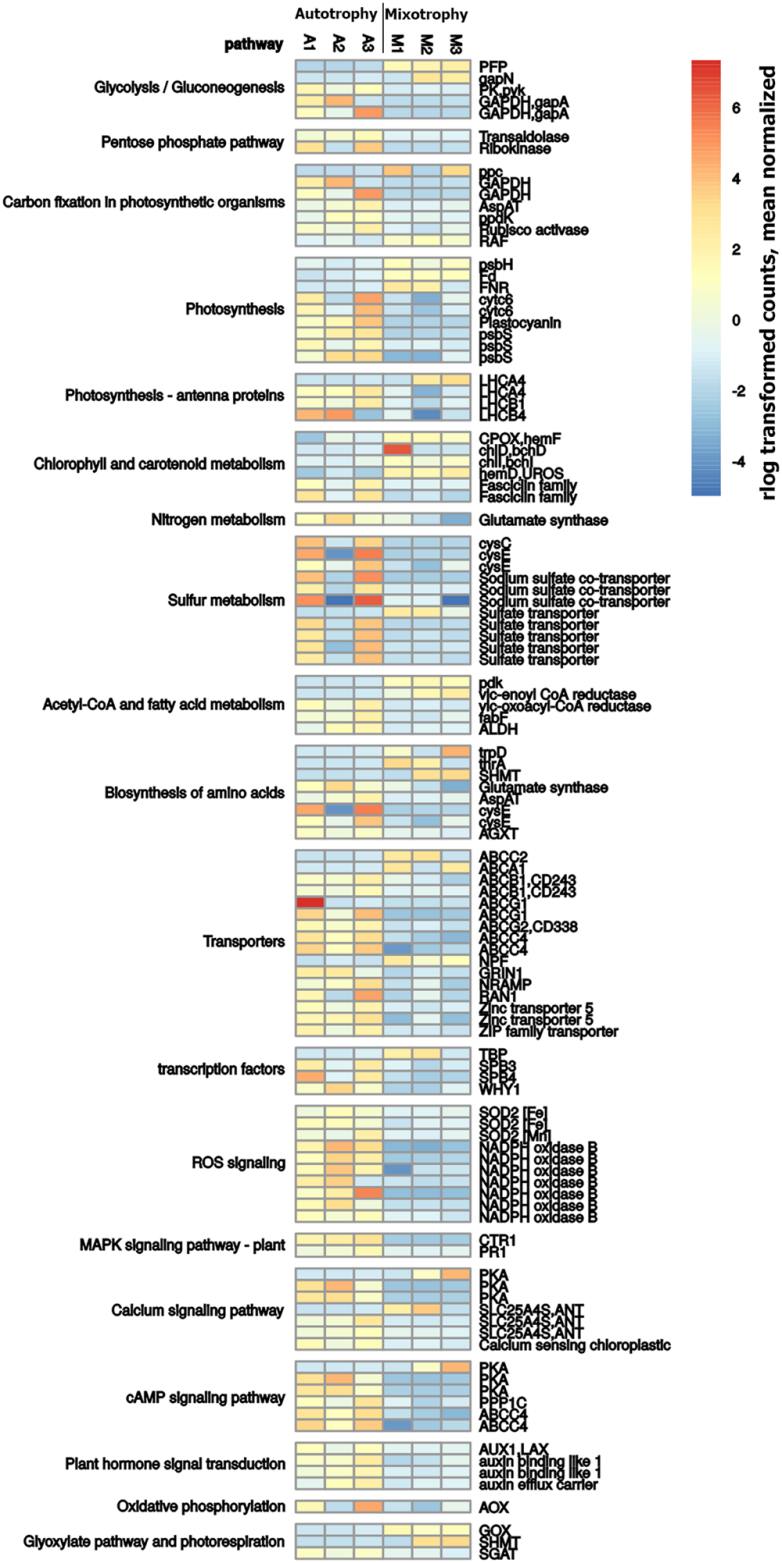
Heat map of selected genes differentially expressed in autotrophy *vs*. mixotrophy. The expression of genes involved in selected pathways modulated in autotrophy *vs*. mixotrophy is shown as log-transformed and mean normalized read counts for each sample analysed (blue, lower abundance; orange, higher abundance).

To investigate the molecular basis of acetate assimilation, the transcriptome of *C*. *sorokiniana* was analyzed in autotrophy and mixotrophy conditions by the mean of RNA-sequencing. Transcriptome was assembled *de novo* identifying 123590 non-redundant contigs (Supplementary Dataset S1), ranging from 224 bp to 16 Kbp and with average length of 1.1 Kbp (Supplementary Figure S1). Transcriptome functional annotation was obtained for 70101 transcripts, of which 53435 were associated to Gene Ontology (GO) Terms (Fig. 3). N50 value of the assembled transcriptome was 1792 bp. To further evaluate the transcriptome quality and completeness, BUSCO analysis was run for a set of 303 universal single-copy genes putatively universally found in eukaryotes as single copies^15^. This analysis identified complete information for 94.4% of orthologs and fragmented information for the 5.0%, only 0.6% are missing, demonstrating a high completeness of the *de novo* assembled transcriptome. Reconstructed 18 S rRNA (TR1035|c8_g4) matched the 18 S rRNA sequences available for *C*. *sorokiniana* UTEX 1230 deposited in NCBI with more than 97% of identity (Supplementary Table S3), confirming the identity of the strain analyzed. Most annotations were obtained by alignment with the publicly available data of *Chlorella variabilis* (Supplementary Figure S2), due to the close evolutive origin of the two species^16,17^. In addition, 15496 annotated sequences were located in KEGG-derived metabolic pathways^18–20^ (Supplementary Figure S2) demonstrating that all the enzymes involved in glycolysis, gluconeogenesis, Krebs cycle, glyoxylate cycle, reductive and non-reductive pentose phosphate cycle and Calvin-Benson cycle were identified in the *de novo* assembled *C*. *sorokiniana* transcriptome. Differential expression analysis revealed that 285 transcripts encoding for 259 different proteins were upregulated in mixotrophy, while 721 transcripts corresponding to 620 proteins were downregulated as compared to autotrophic conditions (Supplementary Dataset S2). Pathway annotation allowed to identify the differentially expressed transcripts involved in relevant biological processes crucial for the algae metabolism among which photosynthesis, chlorophyll and carotenoid metabolism, photorespiratory pathway, carbohydrates, acetyl-CoA, fatty acids and amino acids metabolism, sulphur, nitrogen, phosphate assimilation and transport processes across membrane (Fig. 4).

#### Photosynthesis

**Figure 5:**
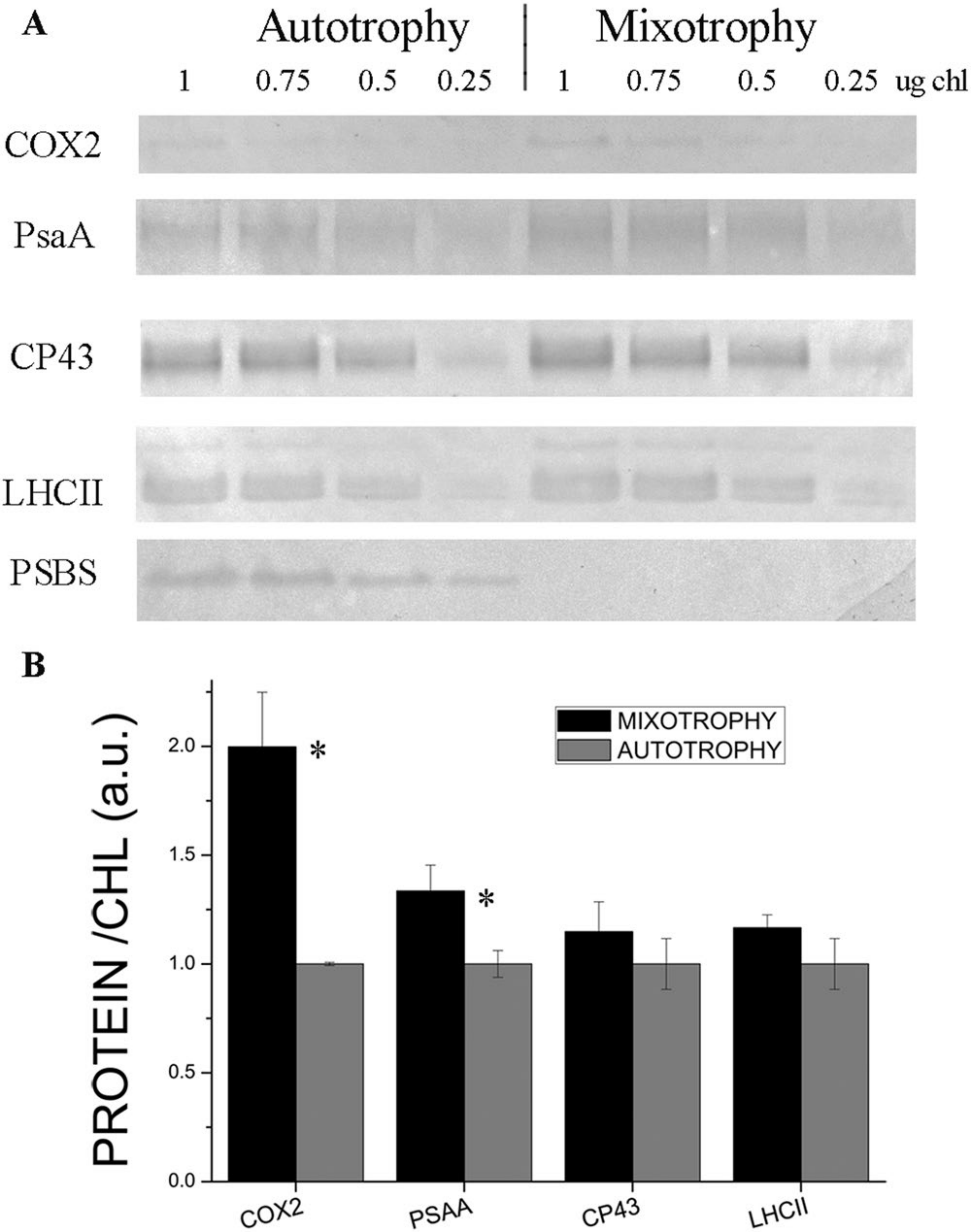
Western blot analysis of chlorophyll binding proteins. PSI, PSII, LHCII and PsbS accumulation in mixotrophy *vs*. autotrophy were investigated by immunoblot analysis. In the case of PSI and PSII their relative accumulation was investigated using antibody recognizing PsaA (subunit of PSI) and CP43 (subunit of PSII). Mitochondrial COX2 subunit was also quantified as a control. Samples were loaded on SDS-PAGE gels in different chlorophyll concentration reported in Panel A. Each immunoblotting analysis was performed loading samples from autotrophy and mixotrophy cultures on the same gel. PsaA, CP43 and LHCII immunoblotting were performed on the same filter cut between 30- and 40 KDa and between 50- and 60 KDa, while PSBS and COX2 immunoblotting analysis where performed on different filters. Quantification of resulting bands were performed by densitometry and normalized to the autotrophy case (Panel B). Significantly different data are indicated (n = 3; *P* < 0.05).

**Figure 6:**
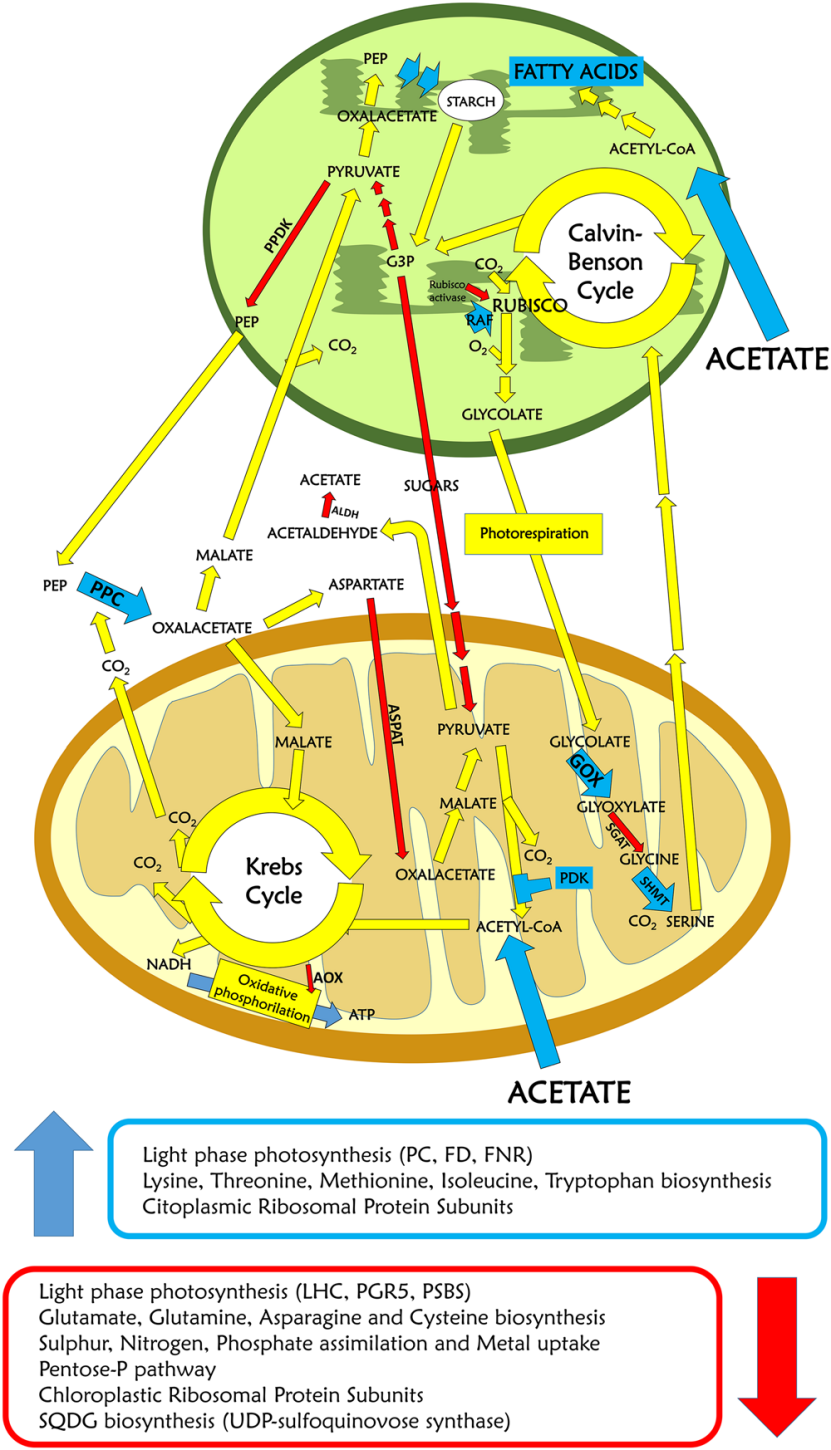
Model of metabolic pathways in autotrophy *vs*. mixotrophy in *C*. *sorokiniana*. Metabolic pathways are reported in yellow if not transcriptionally regulated, in red or blue if down or upregulated in mixotrophy respectively. PEP: phosphoenol-piruvate; RAF: RUBISCO accumulation factor; G3P: Glyceraldehyde 3-phosphate; PDK: pyruvate dehydrogenase kinase; PC: plastocianine; Fd: ferredoxin; FNR: Ferredoxin-NADP^+^ reductase; PPC: phosphoenolpyruvate carboxylase; ALDH: aldehyde dehydrogenase; GOX: glycolate oxidase; SHMT: serine hydroxymethyltransferase; AOX: alternative oxidase; PPDK: pyruvate-orthophosphate dikinase; AspAT: aspartate aminotransferase.

Transcripts coding for LHC subunits homologous to Lhcb4 and LHCII subunits were downregulated in mixotrophy compared to autotrophy, in agreement with the reduced chlorophyll content per cell in the presence of acetate. Similar downregulation of LHC genes has been reported in the case of *C*. *reinhardtii* grown in presence of acetate^21^. However, any downregulation of PSI or PSII core subunits was not observed in mixotrophy, with translational and post-transduction regulative mechanisms likely involved in the overall accumulation of PSII and PSI subunits as previously suggested in the case of *A*. *thaliana*^22^. The accumulation of PSI, PSII and LHCII subunits were thus investigated by immunoblotting (Fig. 5): when loaded with the same amount of chlorophylls, the PSII subunit CP43 and LHCII were detected in a similar amount in both autotrophic and mixotrophic conditions, while the PSI subunit PsaA was slightly increased in mixotrophy. The finding of almost double amount of the mitochondrial subunit COX2 in mixotrophy when SDS-PAGE were loaded at the same amount of chlorophylls nicely fit with the 50% reduction of chlorophyll content per cell in presence of acetate, indicating that in mixotrophy chlorophyll and chlorophyll binding proteins are generally reduced, with a slight increase on a chlorophyll basis of PSI. The increase in PSI content per cell in mixotrophy is likely related to the increased plastoquinone reduction in presence of acetate (Fig. 2D). Differential gene expression analysis indicated also the reduced expression of PSBS subunit in mixotrophy, which was confirmed by western blot analysis, where PSBS accumulation was detected only in autotrophy but not in mixotrophy (Fig. 5). PSBS is a protein subunit crucial for NPQ induction in higher plants, being the sensor of lumenal pH: occurrence of PSBS protein in microalgae has been recently reported for several species, being in *C*. *reinhardtii* transiently induced by high light or UV exposure, even if its functional role in photoprotection is still under debate^23,24^. The similar NPQ traces observed in *C*. *sorokiniana* in presence (mixotrophy) or absence (autotrophy) of PSBS suggest that this protein has a minor role on NPQ induction in this species but could be involved in other photoprotective mechanisms interacting with PSII or LHC subunits. Differently, *C*. *sorokiniana* transcripts homologous to LHCSR proteins, protein subunits involved in NPQ induction in *C*. *reinhardtii*, were not differentially expressed in presence or absence of acetate. Among protein subunits involved photosynthetic electron transport, plastocyanine and cytochrome c6 were downregulated in mixotrophy. It is interesting to note that in mixotrophy downregulation of a PGR5-like subunits was also detected: PGR5 has been reported to be involved in cyclic electron transport around PSI in higher plants and green algae, mediating plastoquinones reduction by Ferredoxin-NADP^+^ reductase (FNR)^25^. Differently, Ferredoxin and FNR subunits were upregulated in mixotrophy: in presence of acetate the increased reducing power derived from acetate consumption causes an overreduction of plastoquinones (Fig. 2), likely inhibiting plastoquinones reduction by cyclic electron transport around PSI but increasing demand of PSI electron acceptor as Ferredoxin and FNR, consistently with the increased PSI content on a chlorophyll basis detected in mixotrophic cells (Fig. 5, Supplementary Figure S3). Differential gene expression was observed in the case of enzymes involved in carbon fixation. Glyceraldehyde 3-phosphate dehydrogenase (phosphorylating) was downregulated in mixotrophy: this is the final enzyme producing glyceraldehyde 3-phosphate in the Calvin-Benson cycle, confirming the crucial role of photosynthates in autotrophy. It is interesting to note that *C*. *sorokiniana* transcriptome revealed the expression of enzymes involved also in a C4-like carbon fixation pathway like either the NAD- or NADP-malic enzyme type or the phosphoenolpyruvate carboxykinase type C4 cycle (Supplementary Figure S4). The presence of a possible C4-like carbon fixation pathway in *C*. *sorokiniana* has been indeed already proposed in the case of the strain LS-2^11^. Among these enzymes, the key subunit involved in carbon fixation is the cytoplasmic phosphoenolpyruvate carboxylase (PPC) was upregulated in mixotrophy, while all the other enzymes putatively involved in a C4-like carbon fixation pathway were similarly expressed in both autotrophy and mixotrophy. Upregulation of PPC in mixotrophy suggests that *C*. *sorokiniana* switches on C4-like carbon fixation in presence of acetate in order to recover CO_2_ released by acetyl-CoA oxidation (Fig. 6). Upregulation of PPC in mixotrophy could also be involved in anaplerotic reaction for oxaloacetate production and CO_2_ recovery in mixotrophic cells characterized by high mitochondrial respiration. Other enzymes putatively involved in C4-like fixation pathway as pyruvate-orthophosphate dikinase (PPDK) and aspartate aminotransferase (AspAT) are instead downregulated in mixotrophy; these enzymes allow respectively for production of phosphoenol-piruvate (PEP) and oxaloacetate from pyruvate and aspartate. PEP and oxaloacetate are intermediates for both C4-like carbon fixation but also for gluconeogenesis and these enzymes could be involved in autotrophy to accumulate PEP and oxaloacetate to be used for glycolysis, gluconeogenesis or other metabolic pathways. Consistently with the increased CO_2_ content in mixotrophy, carbonic anhydrase enzyme resulted to be downregulated. It is interesting to note that in mixotrophy RUBISCO activase was downregulated, while a RUBISCO accumulation factor (RAF) was upregulated: in autotrophy, the relative low CO_2_ content compared to mixotrophy is likely responsible for the needing for increased rubisco activase expression, since this enzyme catalyses the carbamylation of RUBISCO required for its activation^26^, while in mixotrophy RAF upregulation contributes to the assembly of RUBISCO complex to improve carbon fixation. Consistently, increased activity of RUBISCO has been reported in *C*. *sorokiniana* cells grown in mixotrophy in presence of glucose^27^.

#### Carbohydrate metabolism

Among the glycolytic enzymes, fructokinase, glyceraldehyde 3-phosphate dehydrogenase, phosphoglycerate mutase and pyruvate kinase were downregulated in mixotrophy, indicating the crucial role of this metabolic process in absence of acetate, where sugars produced by the dark phase of photosynthesis are used to produce ATP and reducing power. Differently in mixotrophy diphosphate-fructose-6-phosphate-1-phosphotransferase (PFP) and a NADP^+^ dependent glyceraldehyde-3-phosphate dehydrogenase (GapN) were upregulated. PFP, an enzyme found in plants and some bacteria, catalyses the formation of fructose 1,6-bisphosphate from fructose 6-phosphate using inorganic pyrophosphate as the phosphoryl donor, rather than ATP, as in the case of phosphofructokinase (PFK) enzyme: the use of inorganic pyrophosphate makes the reaction reversible, increasing the rate of gluconeogenesis^28^, consistently with the increased starch accumulation observed in mixotrophy. GapN enzyme is a glycolytic enzyme which catalyses an alternative reaction to produce NADPH and glycerate 3-phosphate directly from glyceraldehyde-3-phosphate, without producing the intermediate glyceraldehyde-1,3-bisphosphate: this alternative reaction leads to only one ATP yield for each glyceraldehyde-3-phosphate, but produces NADPH rather than NADH^29^. Phylogenetic analyses and database searches indicated a preferred distribution of GapN in hyperthermophilic Archaea suggesting a role of GapN in metabolic thermoadaptation^29^, which could be related to the thermotolerance of *C*. *sorokiniana*. NADPH in autotrophy is rather produced by the light phase of photosynthesis and by the pentose phosphate pathway: downregulation of a transaldolase and a ribokinase involved in the pentose phosphate pathway were indeed detected in mixotrophy. It is worth to note that in mixotrophy UDP-sulfoquinovose synthase enzyme was downregulated: this enzyme catalyzes the production of UDP-6-sulfoquinovose from UDP-glucose and sulphite, a precursor of sulfolipid sulfoquinovosyl diacylglycerol (SQDG), a key lipid in thylakoid membranes. Downregulation of UDP-sulfoquinovose synthase in mixotrophy could thus be related to the decreased thylakoid accumulation in mixotrophy, consistently with the reduced chlorophyll content observed in presence of acetate.

#### Acetyl-CoA and fatty acids metabolism

Regulative mitochondrial pyruvate dehydrogenase kinase (PDK) was found expressed only in mixotrophy: this serine/threonine kinase inactivates pyruvate dehydrogenase by phosphorylation, inhibiting acetyl-CoA accumulation from pyruvate^30^, as expected in presence of acetate. In addition, an aldehyde dehydrogenase (ALDH) was found downregulated in mixotrophy. ALDH has been reported to be involved in the so called pyruvate dehydrogenase complex (PDHC) bypass pathway, by which pyruvate is converted into acetate which is then imported into chloroplast as acetyl-CoA for fatty acid accumulation^12^: in mixotrophy this enzyme is thus downregulated since acetate is already available in the medium. Only three enzymes involved in fatty acid metabolism were found differentially expressed: a plastid type II fatty acid synthase (FabF), catalysing the first step of fatty acid biosynthesis adding malonyl-ACP to a short fatty acid chain, was detected as downregulated in mixotrophy. This result is consistent with the main role of plastid in autotrophic metabolism and lipid biosynthesis. Interestingly both type I and type II fatty acid synthase enzymes were present in the assembled transcriptome of *C*. *sorokiniana*: type I fatty acid synthase as FAS1 has been reported to be active in the cytoplasm and not present in plants, where usually the plastid type II fatty acid synthase are present. However, the expression of FAS1 enzymes has been already reported in other microalgal species as *Nannochloropsis gaditana*^31^. Other two enzymes involved in the elongation of very long (<16 C) fatty acids longer were found differentially expressed: a very-long-chain 3-oxoacyl-CoA and a very-long-chain enoyl-CoA reductase were downregulated and upregulated respectively in mixotrophy. These enzymes are components of the enzymatic system called elongase that adds two carbons to the chain of long- and very long-chain fatty acids^32^. The different and opposite expression of elongase subunits indicates that the increase lipid content observed in mixotrophy is more influenced on the increased acetyl-CoA availability rather than on the different expression of biosynthetic enzymes.

#### Glyoxylate pathway and photorespiration

Differently from previous works in *C*. *reinhardtii*, isocitrate lyase, the key enzyme for the assimilation of acetate/acetyl-CoA in the glyoxylate pathway^10^ was not upregulated in mixotrophy in *C*. *sorokiniana*. Since glyoxylate production by photorespiratory pathway has been recently proposed to participate to acetate assimilation in *C*. *sorokiniana*, the different enzymes involved in photorespiration were considered, among which glycolate oxidase (GOX), serine hydroxymethyltransferase (SHMT) were upregulated while serine glyoxylate aminotransferase (SGAT) was downregulated. GOX produces glyoxylate from glycolate, which is then the substrate of SGAT which catalyses the interconversion of L-serine and glyoxylate to hydroxypyruvate and glycine (Fig. 6). SHMT catalyses the reversible, conversions of serine to glycine in the mitochondria. Downregulation of SGAT and upregulation of GOX can be related to increase glyoxylate production to be redirected toward the glyoxylate cycle to produce malate upon condensation with acetyl-CoA. The reduced activity of SGAT in mixotrophy would thus cause a reduction in the mitochondria of the substrate of SHMT, glycine, with consequent upregulation of SHMT to complete the photorespiratory cycle.

#### Oxidative phosphorylation

Any differential gene expression was not observed in the case of mitochondrial oxidative phosphorylation, with the exception of alternative oxidase (AOX) which was downregulated in mixotrophy. AOX indeed has been reported not to be involved in acetate metabolism in *C*. *reinhardtii*^33^, and its downregulation in *C*. *sorokiniana* suggests the preferential activation of mitochondrial electron transport via the energy-conserving cytochrome c pathway to produce ATP consuming NADH produced by acetate assimilation.

#### Sulphur, nitrogen, phosphate assimilation and transport systems across membranes

A sodium sulphate co-transporter and adenylylsulphate kinase (CysC) enzyme, involved in sulphate conversion into sulphite, were detected as downregulated in mixotrophy, indicating downregulation of sulphur assimilation pathway in presence of acetate, likely related to the release of acetate caused by sulphur assimilation. Indeed, in plants sulphur assimilation proceeds mainly by cysteine biosynthesis: cysteine production starts from serine which is first acetylated to O-acetyl-serine and then de-acetylated reacting with sulphide to produce L-cysteine releasing acetate. Consistently, in presence of acetate the enzyme serine O-acetyltransferase (CysE) was found downregulated.

Similarly, phosphate transporters and alkane phosphatases, involved in phosphate mobilization, were found downregulated in mixotrophy.

In the case of nitrogen assimilation, the glutamate synthase resulted to be downregulated in mixotrophy, suggesting reduced nitrogen assimilation in this condition: nitrogen assimilation occurs indeed in the chloroplast where nitrate is reduced to nitrite and ammonium, which is then used for the production of glutamate and glutamine. Another enzyme linked to ammonium assimilation, asparagine synthase, was downregulated in mixotrophy. Considering the importance of nitrogen and glutamate for chlorophyll biosynthesis, this result indicates that downregulation of components of nitrogen assimilation is linked with the reduced chlorophyll content per cell observed in mixotrophy. However it is worth to note that in mixotrophy a NRT1/PTR FAMILY (NPF) transporter was found upregulated: NPF is a nitrate or di/tri-peptide transporters which can also transport plant hormones auxin (indole-3-acetic acid), abscisic acid (ABA) and gibberellin (GA), as well as secondary metabolites (glucosinolates)^34^. NPF upregulation could thus be related to the activation of a different nitrate import system or be involved in signalling pathway in mixotrophy, as discussed below. Several ABC transporters resulted to be differentially expressed in autotrophy compared to mixotrophy: ABCB1, ABCC4, ABCG1, ABCG2 were downregulated while ABCA1 and ABCC2 were upregulated in mixotrophy. In mixotrophy, an ionotropic glutamate receptor (GRIN1) was found downregulated: this proteins are ligand gated channels which are involved in particular in Ca^2+^ influx, consistently with the peculiar role of Ca^2+^ in the regulation of cell function in autotrophy. Components involved in iron uptake were downregulated in mixotrophy as in particular the metal transporter NRAMP, the iron binding proteins ferritin and transferrin and multicopper ferroxidase for the oxidation of Fe^2+^ to Fe^3+^. The reduced expression of components involved in iron uptake in mixotrophy is likely linked to the reduced chlorophyll per cell content in presence of acetate, since iron in photosynthetic organisms is mainly located in the thylakoid membranes^35^. It is worth to note that also the copper transporter ATPase RAN1 kinase resulted to be downregulated in mixotrophy: this kinase is also a component of the ethylene signalling pathway^36^. Moreover a zinc transporter and a ZIP family transporter^37^ were observed downregulated in mixotrophy, confirming the general reduced expression in mixotrophy of genes involved in nutrient assimilation and metal uptake.

#### Chlorophyll and carotenoid metabolism

Chlorophyll content per cell was reduced in presence of acetate (Supplementary Table S1). However, transcripts encoding for uroporphyrinogen-III synthase, coproporphyrinogen III oxidase and magnesium chelatase, which leads to production of the chlorophyll precursor Mg-protoporphyrin-IX from glycine were upregulated in mixotrophy. These results suggest that the regulation of nitrogen and iron uptake rather than differential expression of chlorophyll biosynthetic enzyme is at the base of the control of chlorophyll content per cell in *C*. *sorokiniana*. In the case of carotenoid biosynthetic pathway, a carotenoid oxygenase was downregulated in mixotrophy, even if the function of this enzyme remains unclear. Moreover, in mixotrophy two transcripts coding for astaxanthin binding fasciclin family proteins were detected as downregulated: these proteins were reported as water soluble protein that bind carotenoids and in particular astaxanthin, being involved in microalgae in the resistance to photo-oxidative stress^38^. Astaxanthin was not detected in *C*. *sorokiniana* in these conditions (Supplementary Table S1), even if the enzymes CrtO and CrtZ for its production were identified in the annotated transcriptome. More stressful conditions could be required for astaxanthin significant production with a putative role of astaxanthin binding fasciclin family proteins for its accumulation^39^.

#### Amino acids metabolism

As discussed above, glutamate synthetase, asparagine synthase, serine O-acetyltransferase and aspartate aminotransferase enzymes involved respectively in glutamate/glutamine, asparagine and cysteine biosynthesis and aspartate de-amination to produce oxaloacetate were found downregulated in mixotrophy. In addition, transcripts encoding for a serine-glyoxylate transaminase were found downregulated in presence of acetate: this enzyme catalyses the production of glycine and 3-hydroxypyruvate from serine and glyoxylate and its downregulation is consistent with the increased requirement of glyoxylate in mixotrophy for acetate assimilation and with the reduced glycine demand, considering the use of glycine as the precursor for chlorophyll biosynthesis. Differently a chloroplastic-like bifunctional aspartokinase homoserine dehydrogenase (ThrA) was upregulated in presence of acetate: this enzyme was found in bacteria and plant chloroplasts, catalysing the first and third steps of the aspartate pathway, by which aspartate is used as a precursor for lysine, threonine, methionine and isoleucine. In addition, an anthranilate phosphoribosyltransferase (trpD) enzyme was upregulated in mixotrophy: this enzyme catalyses the accumulation of the precursors for tryptophan biosynthesis. Increased expression of enzymes involved in the biosynthesis of tryptophan, lysine, threonine, methionine and isoleucine could be the related to the increased protein content per cell observed in mixotrophy. Moreover, consistently with the increase protein accumulation observed in mixotrophy, several ribosomal protein subunits were upregulated in presence of acetate. Only in the case of ribosomal chloroplast subunit RP-S16 and RP-LE30 downregulation was observed in mixotrophy, consistently with the reduced chlorophyll content and reduced accumulation of chlorophyll binding subunits observed in presence of acetate.

#### Transcription factor and signalling

Three transcription factors were found differentially expressed in autotrophy compared to mixotrophy: a transcription initiation factor TFIID TATA-box-binding protein (TBP) were upregulated in mixotrophy, while two Squamosa promoter binding protein (SPB3 and SPB4) and a Whirly transcription factor were found downregulated in mixotrophy. TBP is a general transcription factor involved in the RNA polymerase II preinitiation complex^40^, while SBP and a Whirly transcription factor are transcription factors commonly found in plants. SBP transcription factors play important roles among others in flower and fruit development, plant architecture and response to hormones^41^, while Whirly transcription factors were reported to be involved in plant defense^42^, chloroplast biogenesis^43^ and plastid genome stability^44^. Downregulation of SBP and Whirly transcription factors in mixotrophy could be related to the reduced chlorophyll content photosynthetic proteins per cell and a reduced autotrophic metabolism observed in presence of acetate. Several transcripts involved in signalling were found differentially expressed in autotrophy compared to mixotrophy. The MAP kinases PKA, RAN1, CTR1 and PR1 were found downregulated in mixotrophy while a different transcript for PKA, CTR1 and LZK were upregulated in mixotrophy (Supplementary Table S3). It is interesting to note that PR1 kinase in *A*. *thaliana* has been reported to be involved in biotic stress defense being a component of the salicylic acid signalling^45^, while CTR1 and RAN1 are component of the ethylene signalling pathway in higher plants^36^. In autotrophy, an increased expression of protein involved in auxin response as the auxin transporter AUX1, an auxin efflux carrier and an auxin binding protein were also detected suggesting a possible ethylene/auxin dependent modulation of gene expression in autotrophy. Interestingly a cyclin dependent kinase and G2/mitotic-specific cyclin-B were detected upregulated in mixotrophy, suggesting a possible role of cyclin in the increased cell density yielded in presence of acetate (Supplementary Table S4). Other serine/threonine kinase and phosphatase are upregulated or downregulated in mixotrophy, indicating specific signalling pathways and phosphorylation processes in these conditions (Supplementary Table S4). It is interesting to note the down-regulation of a chloroplast calcium sensing (CAS) protein and a calcium-dependent kinase in mixotrophy, consistently with the downregulation of putative calcium influx channel GRIN1 in presence of acetate as discussed above. CAS has been reported in *C*. *reinhardtii* to be involved in the regulation of photoacclimation and calcium dependent regulation of photosynthetic cyclic electron transport^46,47^, which is indeed enhanced in autotrophy compared to mixotrophy. Interestingly a subunit of superoxide-generating NADPH oxidase was found downregulated in mixotrophy: this enzyme produces the reactive oxygen species superoxide and it has been reported to be involved in reactive oxygen species dependent signalling mechanisms^48^. Similarly, two transcripts coding for superoxide dismutase, which catalyzes superoxide scavenging was downregulated in the same mixotrophic conditions, indicating fine regulation of the superoxide concentration as a possible signal for acclimation to different growth conditions.

## Discussion

The study describes the impact of acetate availability on gene expression and photosynthetic properties of *C*. *sorokiniana*. The availability in the medium of a reduced carbon source as acetate increased biomass yield, cell density and daily productivity of *C*. *sorokiniana* cells compared to autotrophic growth. Increase in biomass yield and productivity in mixotrophy was related mainly to an increase of starch and lipid content per cell, even if total protein content was also increased. Photosynthetic traits were not significantly affected in mixotrophy compared to autotrophy: however, upregulation of genes coding for electron acceptors downhill plastoquinones pool as plastocianine, ferrodoxin and FNR, and downregulation of PGR5-like subunit suggests increased electron transport from plastoquinones to NADPH and reduced cyclic electron transport across PSI. Increased reduction of plastoquinone pool in presence of acetate is indeed related to increase transferring of reducing power from mitochondria to the chloroplast, as a consequence of increased NADH production during acetate assimilation. The reduced chlorophyll content per cell observed in mixotrophy was not controlled by differential gene expression of chlorophyll biosynthetic enzymes but rather by downregulation of protein subunits involved in nitrogen assimilation, glycine biosynthesis, iron uptake and accumulation of thylakoid lipid SQDG. Assimilation of acetate has been reported in several organisms to be linked to glyoxylate cycle^9,10^ and photorespiration^49^, by which glyoxylate is produced. The advantage of glyoxylate cycle toward acetate assimilation is the production of NADH without decarboxylation of isocitrate occurring in the Krebs cycle with the loss of two CO_2_ molecules. However, the presence of acetate did not induce in *C*. *sorokiniana* any upregulation of glyoxylate cycle enzymes but only caused an increased expression in mixotrophy of the enzymes GOX and serine hydroxymethyltransferase (SHMT), which are both involved in the photorespiratory pathway (Fig. 6)^49^. Acetul-CoA produces by acetate assimilation is thus likely consumed in the Krebs cycle or through the glyoxylate produced by a photorespiratory-like pathway: it is important to note that assimilation of acetate through these pathways is accompanied by CO_2_ release by isocitrate decarboxylation or by the activity of the SHMT enzymes respectively, increasing the relative CO_2_ concentration in mixotrophic cells. Increased CO_2_ production in mixotrophic conditions is confirmed by the downregulation in presence of acetate of several genes commonly induced in *C*. *reinhardtii* by relative low CO_2_ concentration as carbonic anhydrase, RUBISCO activase or components of carbon contrating mechanism (Fig. 4)^26,50^. *De novo* transcriptomes reported in this or previous works^11^, demonstrate in *C*. *sorokiniana* the presence and expression of genes involved in C4-like carbon fixation pathway: interestringly, the key CO_2_ fixing enzyme PPC is upregulated in mixotrophy (Fig. 4). These findings suggest that, carbon loss due to acetate oxidation is reduced by the activation in mixotrophy of an alternative carbon fixation pathway by PPC. This strategy allows to maximize the energetic yield of acetate assimilation, reducing the loss of carbon atoms. C4-like carbon fixation pathway has been already reported in the case of diatoms^51^, suggesting a peculiar properties of some unicellular microalgae to improve CO_2_ assimilation using different pathway in parallel with the Calvin-Benson cycle.

In conclusion, a fine regulation of cellular metabolism is induced by the availability of acetate in the growth medium. The metabolism shift was characterized by the downregulation of glycolysis, pentose phosphate pathway and acetyl-CoA production from pyruvate, while glyoxylate production, biosynthesis of several amino acids and protein translation are increased. Acetate induces also an increase of lipid accumulation which however is not directly related to differential expression of biosynthetic genes. Three transcription factors were identified as differently expressed in autotrophy *vs*. mixotrophy with TBP upregulated in mixotrophy and the plant specific SBP and Whirly transcription factors downregulated in mixotrophy. These transcription factors are putatively responsible for the different gene expression herein reported, even if the activation/inhibition of other transcription factor cannot be excluded. Several kinases and phosphatases were indeed differently expressed in presence or absence of acetate, which could be involved in the regulation of gene expression. Components involved in ethylene, auxin, salicylic acid and calcium signalling were downregulated in mixotrophy, pointing for a complex network of regulation of gene expression and cell functions. Even if further work is necessary, these signalling components may have a role in the regulation of gene expression in *C*. *sorokiniana* under autotrophic condition and possibly in other unicellular microalgae, similar to what described in multicellular higher plants.

## Methods

### Microalgae cultivation

*C*. *sorokiniana* UTEX 1230 cells were grown in 1 L airlift photobioreactors in BG11 medium at 450 µmol m^−2^s^−1^ with day/night cycles of 16/8 hours with CO_2_ addition “on demand” on the base of the pH of the medium as described in^52^, with the addition of acetate (1 gr/L) in the case of mixotrophic cultivation. Daily cell counts and dry weight determination were performed as described in^52^.

### Photosynthetic parameters

Fluorescence parameters (Fv/Fm, NPQ) were measured by WALZ PAM-100 fluorometer as described in^52^. Electrochromic shift (ECS) measurements at 520 nm were performed using a Joliot-type spectrophotometer JTS-10 (BioLogic) as described in^14^. Oxygen evolution curves were measured as described in^52^.

### RNA extraction and RNA seq analysis

RNA was extracted from three independent biological replicates of each culture condition at exponential phase using a modified TRIzol SIGMA-ALDRICH protocol. Cells were disrupted by glass beads (Micro-organism lysing VKmix, Bertin Technologies). RNA samples were further purified with the SIGMA Spectrum Plant Total RNA kit including a DNAse treatment step. RNA quality and quantity were determined using a Nanodrop 2000 spectrophotometer (Thermo Scientific, Wilmington, DE) and a Bioanalyzer Chip RNA 7500 series II (Agilent, Santa Clara, CA). Directional RNA-seq library preparation was performed starting from 1 ug total RNA using the TruSeq RNA Sample Prep Kit v2 (Illumina Inc., San Diego, CA, USA) after capturing poly-adenylated transcripts. Library quality was assessed with a High Sensitivity DNA Kit on a 2200 Tape Station (Agilent, Wokingham, UK). Libraries were sequenced with an Illumina HiSeq. 1000 sequencer (Illumina Inc., San Diego, CA, USA) generating ~22 million 100-bp paired-end reads per sample. Low-quality reads (>50 bases with quality <7 or >10% undetermined bases) and putative PCR duplicate reads were removed and Illumina TruSeq adapter sequences were clipped. Low-quality bases at read ends were trimmed (minimum quality 16, minimum read length 50 bp) with cutadapt (http://code.google.com/p/cutadapt/).

### Transcriptome de-novo assembly and differential expression analysis

*De novo* transcriptome assembly was carried out with the Trinity (v. 2.0.6) software using reads of all samples as input with the following parameters:–seqType fq,–max_memory 128 G,–CPU 20,–min_contig_length 200,–jaccard_clip,–normalize_reads –normalize_by_read_set,–SS_lib_type RF, and a default k-mer value of 25. Abundance of each transcript was calculated using the align_and_estimate_abundance.pl script from Trinity software 2.0.6. Default settings were used except for the options–est_method RSEM–aln_method bowtie–trinity_mode—prep_reference—SS_lib_type RFFR. RSEM version 1.2.2919 was used to estimate the abundance of each transcript. The row count matrix was at first filtered removing all the very low expressed genes showing a mean expression value below 10 reads count across all the samples. The identification of the differentially expressed genes was performed using the negative binomial distribution-based method implemented in EdgeR^53^. The counts were normalized to take into account the different sequencing depth using the TMM (Trimmed Mean of M-value) method implemented in EdgeR. Genes with a *p-value* lower or equal 0.05, after false discovery rate correction, were considered significantly differentially expressed. Differential expression analysis was performed using the negative binomial distribution-based method implemented in DESeq on the summed read counts per transcript^54^.

### Transcriptome functional annotation

The transcriptome functional annotation was performed by means of a similarity search against the non-redundant protein database (NCBI) using Blastp and setting an evalue threshold of 1e-5. Further annotation were retrieved using InterProscan5^55^ against several different databases (PROSITE patterns, PRINTS, PFAM, PRODOM, SMART, TIGRFAM and PANTHER) for the identification of conserved protein domains and functional annotation. Gene Ontology annotations were assigned running Blast2GO 2.6.0^56^ on the blast and InterProscan results. Sequences were also analysed by KAAS (KEGG Automatic Annotation Server) platform to obtain KO annotation^18–20^. Transcripts differently expressed with KO annotation were visualized by KEGG Mapper platform (http://www.kegg.jp/kegg/tool/map_pathway.html), while the remaining transcripts functionally annotated were manually inspected by retrieving the function of the closest homolog gene. List of transcripts identified in the *de novo C*. *sorokiniana* assembled transcriptome with functional annotation, where retrieved, are reported in Supplementary data, Data file S1. Trascriptome completeness was evaluated by BUSCO analysis using as a reference a dataset of 303 universal single-copy genes putatively universally found in eukaryotes as single copies^15^.

### Statistical analysis

Descriptive statistical analysis with mean and standard deviation were applied for all the data reported except for differential gene expression analysis. Differential gene expression was considered significant when the *p-value* associated to the comparison autotrophic vs. mixotrophic was lower or equal 0.05, after the false discovery rate correction implemented in the DESeq algorithm. The non-parametric Mann-Whitney was applied to compare the impact of autotrophic vs. mixotrophic conditions, unless otherwise stated. All the analyses were performed using OriginPro 8 software. Differences of *P* < 0.05 were considered significantly different.

### Data availability

All the data generated during and/or analyzed during the current study are available from the corresponding author on reasonable request. All next-generation sequencing data and contigs assembled from Illumina reads are available in the NCBI Bioproject PRJNA416862. In the Supplementary Dataset S3 the sequences of all transcripts identified in this work are reported.

## Electronic supplementary material

Supplementary Table S1-4 and Figure S1-4

Dataset 1

Dataset 2

Dataset 3
