# Supplementary material for: Molecular basis of autotrophic vs mixotrophic growth in Chlorella sorokiniana
Source: Sci Rep. 2018 Apr 24;8:6465. doi: 10.1038/s41598-018-24979-8 (PMC5915390; doi:10.1038/s41598-018-24979-8)
Supplement: Supplementary file 1 — Supplementary Table S1-4 and Figure S1-4 [file 41598_2018_24979_MOESM1_ESM.pdf]

Molecular basis of autotrophic vs mixotrophic growth in *Chlorella sorokiniana*

Michela Cecchin[1], Salvatore Benfatto[1], Francesca Griggio[1], Alessia Mori[1], Stefano Cazzaniga[1], Nicola Vitulo[1], Massimo Delledonne[1], Matteo Ballottari[1*]

[1] Dipartimento di Biotecnologie, Università di Verona, Strada Le Grazie 15, 37134 Verona, Italy

**Corresponding author. Email: matteo.ballottari@univr.it*

Supplementary data

Table S1. Pigment analysis of *C. sorokiniana* cells in autotrophy *vs.* mixotrophy
Chlorophyll content per cell is reported as pg of chlorophyll. Pigment data are reported normalized to 100 chlorophylls. Chl a/b: chlorophyll a/b; Car: carotenoid; Nx: neoxanthin; Vx: violaxanthin; Ax: anteraxanthin; Lut: Lutein; β-Car: β-carotene. Standard deviation (s.d.) are reported (n = 6).

	Chl/cell	Chl a/b	chl/car	Chl a	Chl b	Nx	Vx	Ax	Lut	β-Car
**Mixotrophy**	1.10E-07	2.71	3.50	73.04	26.96	5.24	1.76	0.53	18.13	2.88
s.d.	*1.43E-08*	*0.17*	*0.01*	2.58	1.43	*0.26*	*0.03*	*0.13*	*0.61*	*0.14*
**Autotrophy**	2.03E-07	3.01	3.53	75.08	24.92	5.24	1.87	0.47	18.00	2.79
s.d.	*3.19E-08*	*0.50*	*0.16*	6.86	3.42	*0.11*	*0.13*	*0.05*	*1.03*	*0.02*

Table S2. Photosynthesis and respiration rates. O_2 evolution/consumption were measured with a Clark-type oxygen electrode. Standard deviation are reported (n = 4).

	AUTOTROPHY	*s.d*	MIXOTROPHY	*s.d*
**Pmax (μmol O_2 h^{-1} mg Chl^{-1})**	128.60	*3.67*	128.36	*8.26*
**Half-saturation intensity (μmol m^{-2} s^{-1})**	135.17	*22.93*	115.22	*27.11*
**Respiration (μmol O_2 h^{-1} $cells^{-1}$)**	4.82E-09	1.43E-09	6.20E-09	9.84E-10
**Respiration (μmol O_2 h^{-1} mg Chl^{-1})**	23.75	8.55	53.61	11.28

Table S3: Identification of 18S transcripts in C. sorokiniana denovo assembled genome using C. sorokiniana UTEX1230 18S sequences available at NCBI

***C. sorokiniana* UTEX 1230 sequence**	**First sequence aligned in denovo *C. sorokiniana* transcriptome**	**E-value**	**Identities**	**Gaps**
KR904895.1	TR1035|c8_g4_i1	0	1110/1140 (97%)	10/1140 (1%)
KJ676112.1		0	Identities = 1404/1436 (98%)	11/1436 (1%)
KP645225.1		0	737/739 (99%)	1/739 (0%)

Table S4: Putative regulative phosphatases and kinases differently regulated in mixotrophy compared to autotrophy in *C. sorokiniana*

	Transcript	logFC	Description
**DOWNREGULATED IN MIXOTROPHY**			
**PHOSPHATASES**	TR7705|c0_g1_i2	-4.34233	Phytochrome-associated serine threonine-phosphatase
	TR7716|c0_g8_i1	-1.85008	phosphatase 1
	TR7716|c0_g8_i3	-3.9312	phosphatase 1
	TR51265|c0_g1_i12	-1.62184	ser thr phosphatase family
	TR51284|c0_g1_i3	-7.73373	probable serine threonine phosphatase 2A regulatory subunit B delta isoform X2
	TR13958|c1_g2_i1	7.875484	phosphoinositide phosphatase SAC9
**KINASES**	TR9072|c0_g1_i2	-11.4016	Serine threonine- kinase CTR1
	TR1026|c3_g5_i8	-9.20802	Serine threonine- kinase CTR1
	TR1066|c3_g2_i16	-10.2851	cyclic nucleotide dependent kinase
	TR1066|c3_g2_i17	-9.15322	cyclic nucleotide dependent kinase
	TR43814|c1_g2_i4	-10.8645	Serine threonine- kinase CTR1
	TR43853|c0_g2_i1	-2.46651	probable receptor kinase At5g39020
	TR44339|c0_g1_i1	-7.78603	Serine threonine- kinase CTR1
	TR45584|c0_g4_i9	-3.22761	Serine threonine- kinase
	TR50242|c0_g1_i3	-3.82414	serine threonine- kinase
	TR50242|c0_g1_i4	-2.33285	serine threonine- kinase
	TR53297|c1_g1_i17	-9.12487	Calcium-dependent kinase 29
	TR9072|c0_g1_i2	-11.4016	Serine threonine- kinase CTR1
**UPREGULATED IN MIXOTROPHY**			
**PHOSPHATASES**	TR11354|c0_g1_i1	7.287051	phosphatase 1 regulatory subunit 7
	TR13958|c1_g2_i1	7.875484	phosphoinositide phosphatase SAC9
	TR24888|c0_g1_i2	7.714205	phosphoinositide phosphatase SAC6-like
**KINASES**	TR17426|c0_g2_i3	2.274882	Serine threonine- kinase CTR1
	TR2167|c0_g1_i1	8.781701	Serine Threonine kinase
	TR2977|c0_g1_i1	4.872873	cyclin dependent kinase
	TR4032|c0_g5_i10	7.556271	serine threonine- kinase receptor R831
	TR43839|c0_g1_i5	8.889553	serine threonine- kinase ATR
	TR45177|c0_g1_i20	3.200082	serine threonine- kinase
	TR45177|c0_g1_i32	8.550787	serine threonine- kinase
	TR45657|c5_g6_i5	7.554033	serine threonine- kinase receptor R831
	TR51340|c1_g3_i12	7.626945	probable serine threonine- kinase At1g09600
	TR981|c0_g1_i4	9.336607	Serine threonine- kinase CTR1

Figure S1. *De novo* assembly of C. sorokiniana transcriptome, distribution of transcript lengths among the different sequences.

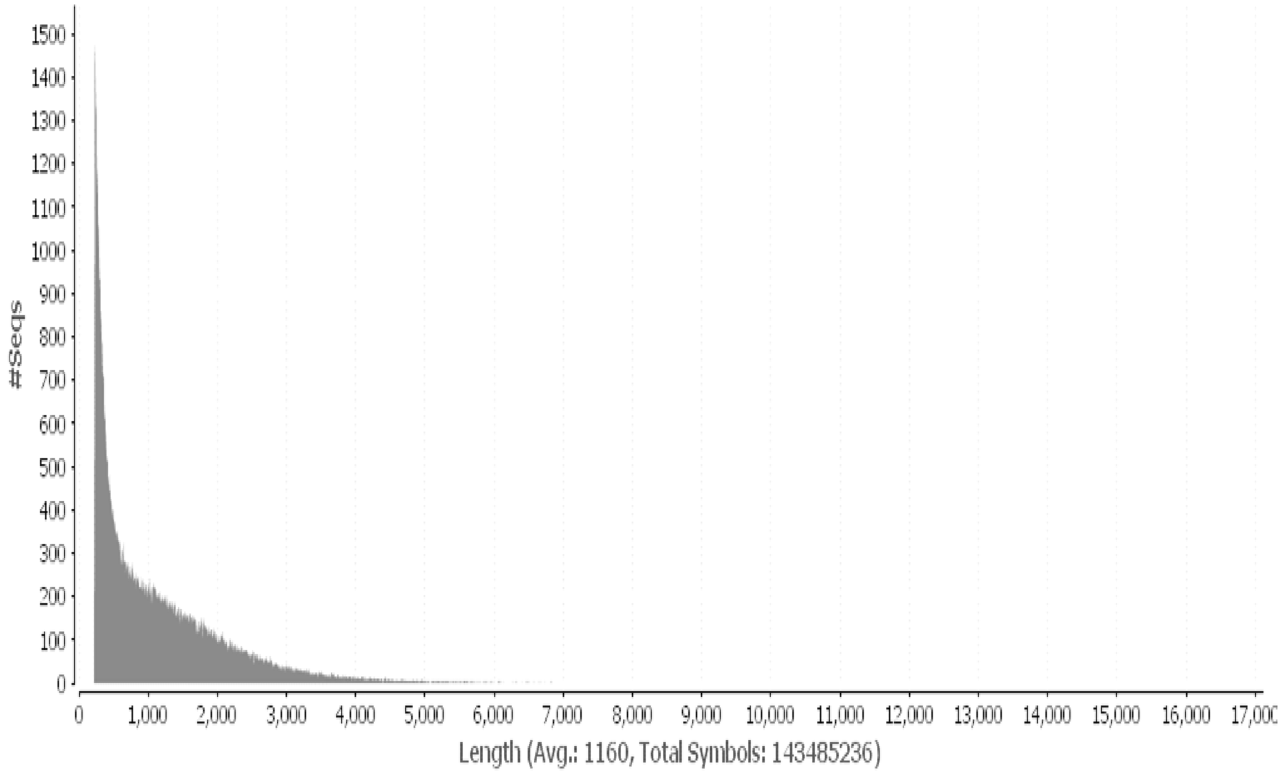

Figure S2. Functional annotation of C. sorokiniana transcriptome. Panel A. data distribution among total transcripts with Gene Ontology (GO) and KEGG Orthology (KO) annotation. Panel B: number of sequences with a number of GO terms reported on the X-axis. Panel C: Distribution of annotated sequences among different species

A

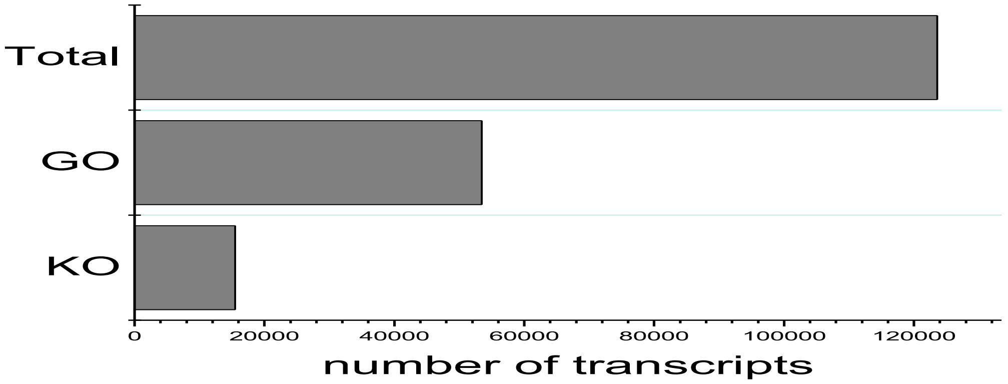

B

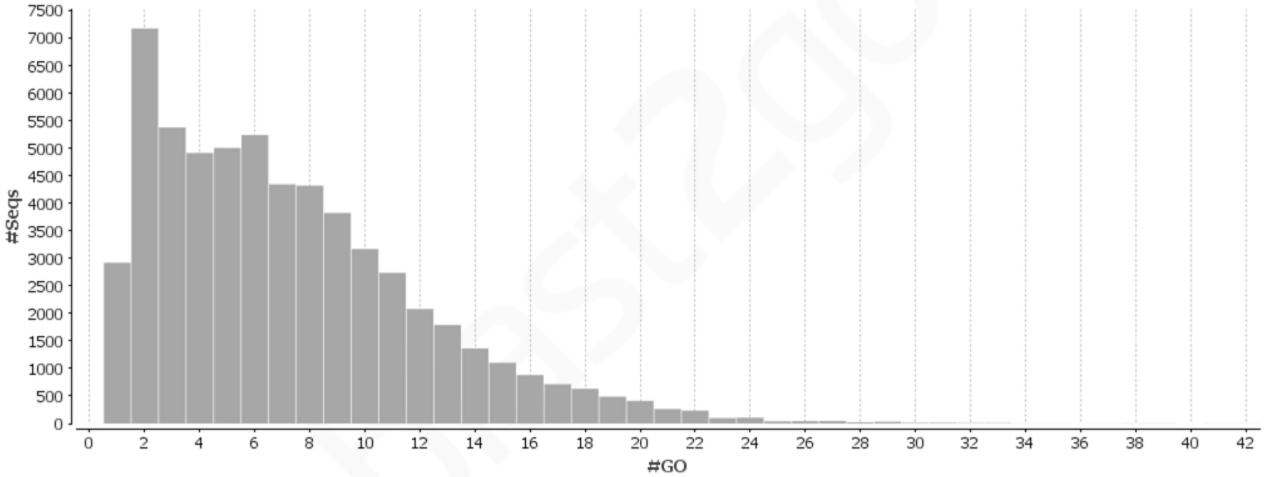

C

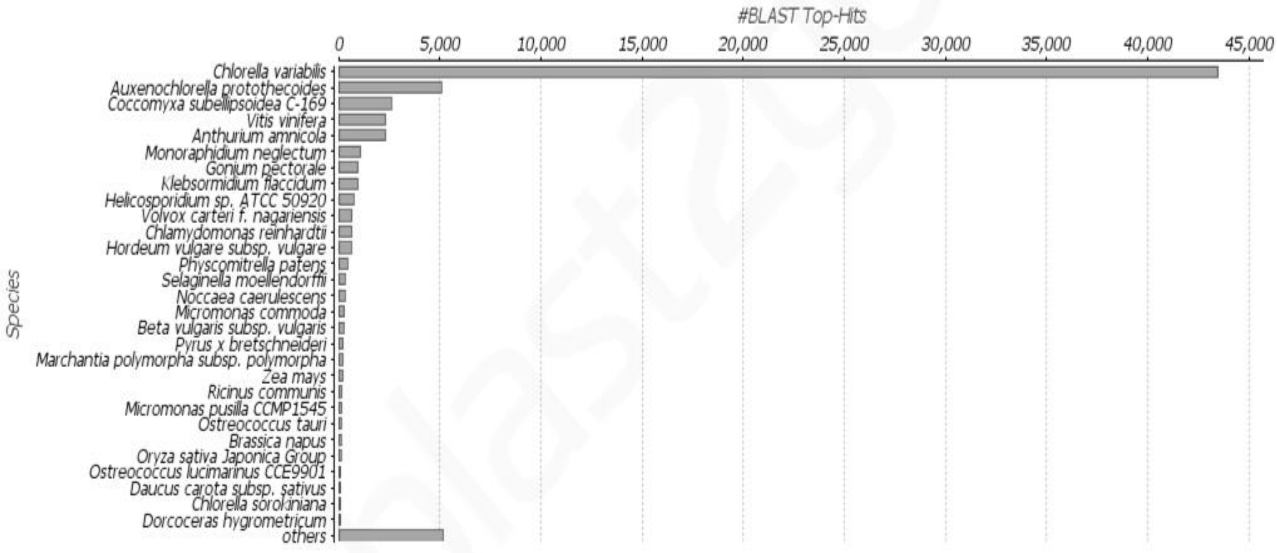

Figure S3. Western blot analysis on photosynthetic subunits. SDS-PAGE gels were transferred on nitrocellulose filters which were cut between 30 and 40 KDa and between 50 and 60 KDa according to the migration of prestained molecular marker (NIPPON Genetics Europe, PINK Presatined Protein Ladder). Immunoblotting analysis using LHCII, PSBS and COX2 specific antibodies were performed on the filters with protein at lower apparent MW, CP43 on the filters with proteins at intermediate (50-40 KDa) apparent MW, and PsaA on the filters with proteins at higher apparent MW.

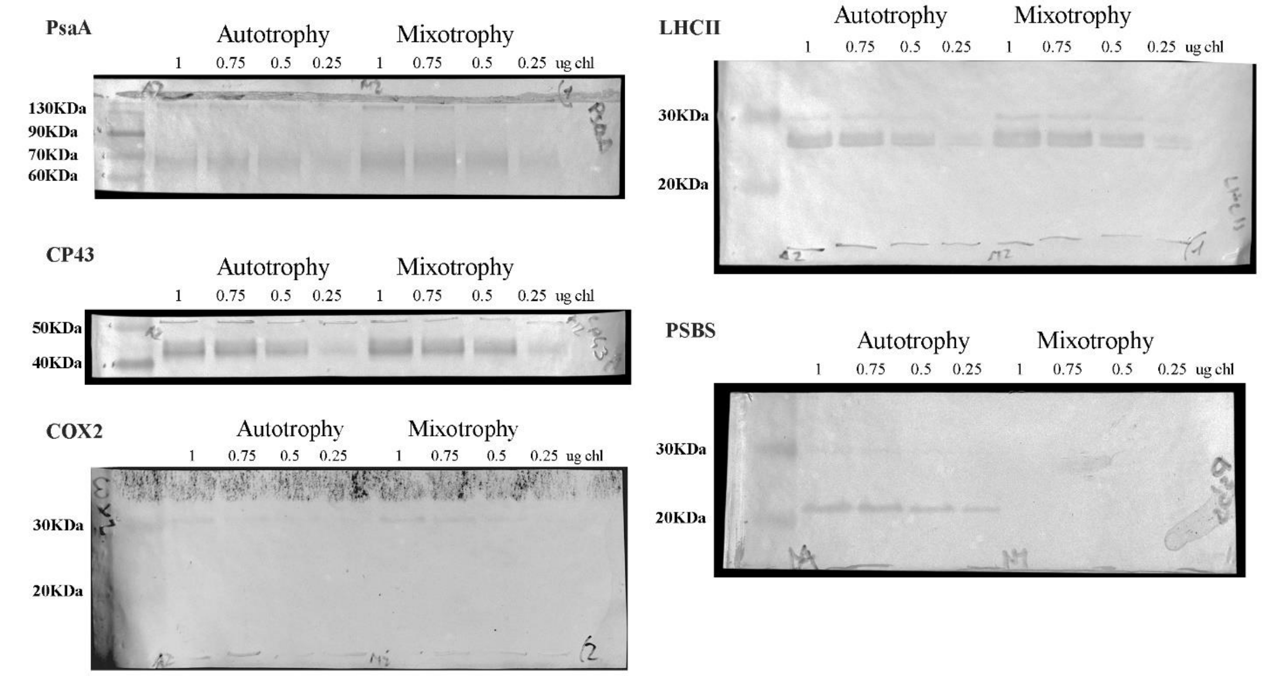

Figure S4. Carbon fixation pathways in *C. sorokiniana*. Carbon fixation pathways visualized on the base of KEGG Map Pathway (http://www.kegg.jp/kegg/tool/map_pathway.html)[1-3]. Metabolic reactions for which catalysing enzymes were detected in the *C. sorokiniana* transcriptome are indicated in yellow in the case of enzymes not differentially expressed, blue and red in case of enzymes respectively upregulated and downregulated in mixotrophy. PPC: phosphoenolpyruvate carboxylase; PPDK: pyruvate-orthophosphate dikinase; AspAT: aspartate aminotransferase; GAPDH: Glyceraldehyde 3-phosphate dehydrogenase (phosphorylating)

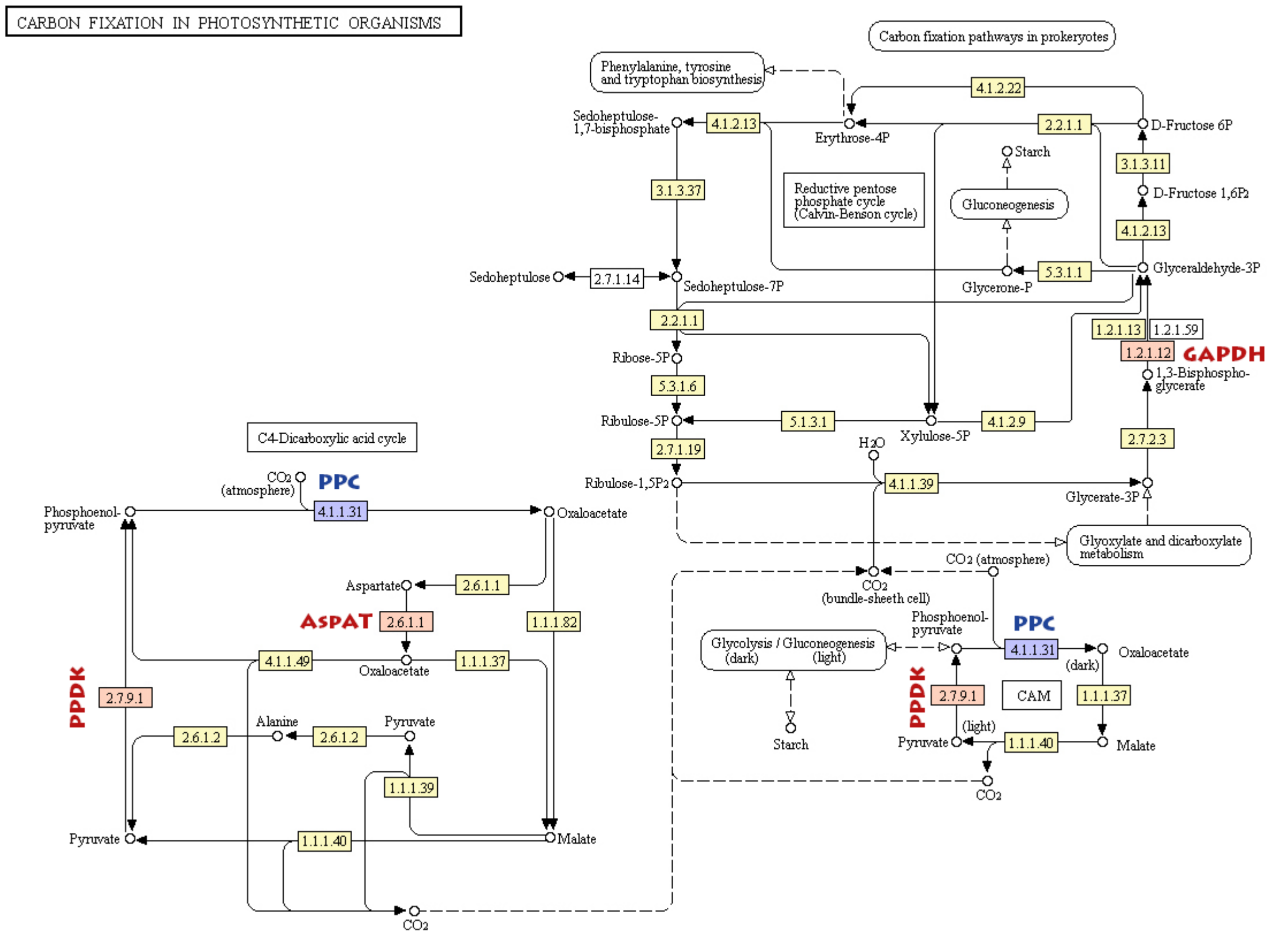

Dataset S1: List of *C. sorokiniana* transcripts identified in the *de novo* assembled transcriptome with functional annotation retrieved by blast2go.

Dataset S2: List of *C. sorokiniana* transcripts differently regulated in mixotrophy compared to autotrophy.

Dataset S3: FASTA sequences of all transcripts identified in this work in *C. sorokiniana* cells grown in autotrophy or mixotrophy.

REFERENCES

1 Kanehisa, M., Furumichi, M., Tanabe, M., Sato, Y. & Morishima, K. KEGG: new perspectives on genomes, pathways, diseases and drugs. *Nucleic Acids Res* **45**, D353-D361, doi:10.1093/nar/gkw1092 (2017).

2 Kanehisa, M., Sato, Y., Kawashima, M., Furumichi, M. & Tanabe, M. KEGG as a reference resource for gene and protein annotation. *Nucleic Acids Res* **44**, D457-462, doi:10.1093/nar/gkv1070 (2016).

3 Kanehisa, M. & Goto, S. KEGG: kyoto encyclopedia of genes and genomes. *Nucleic Acids Res* **28**, 27-30 (2000).
